# Dual-Path Convolutional Neural Network with Squeeze-and-Excitation Attention for Lung and Colon Histopathology Classification

**DOI:** 10.3390/jimaging11120448

**Published:** 2025-12-14

**Authors:** Helala AlShehri

**Affiliations:** Computer and Information Technology Department, Jubail Industrial College, P.O. Box 10099, Jubail Industrial City 31961, Saudi Arabia; shehrihel@rcjy.edu.sa

**Keywords:** explainable AI, interpretable deep learning, computer vision, histopathology, lung cancer, colon cancer, dual-path CNN, SE attention, Grad-CAM, integrated gradients

## Abstract

Lung and colon cancers remain among the leading causes of cancer-related mortality worldwide, underscoring the need for rapid and accurate histopathological diagnosis. Manual examination of biopsy slides is often time-consuming and prone to inter-observer variability, which highlights the importance of developing reliable and explainable automated diagnostic systems. This study presents DPCSE-Net, a lightweight dual-path convolutional neural network enhanced with a squeeze-and-excitation (SE) attention mechanism for lung and colon cancer classification. The dual-path structure captures both fine-grained cellular textures and global contextual information through multiscale feature extraction, while the SE attention module adaptively recalibrates channel responses to emphasize discriminative features. To enhance transparency and interpretability, Gradient-weighted Class Activation Mapping (Grad-CAM), attention heatmaps, and Integrated Gradients are employed to visualize class-specific activation patterns and verify that the model’s focus aligns with diagnostically relevant tissue regions. Evaluated on the publicly available LC25000 dataset, DPCSE-Net achieved state-of-the-art performance with 99.88% accuracy and F1-score, while maintaining low computational complexity. Ablation experiments confirmed the contribution of the dual-path design and SE module, and qualitative analyses demonstrated the model’s strong interpretability. These results establish DPCSE-Net as an accurate, efficient, and explainable framework for computer-aided histopathological diagnosis, supporting the broader goals of explainable AI in computer vision.

## 1. Introduction

Lung and colon, or colorectal (LC), cancers are among the leading causes of cancer-related mortality worldwide, underscoring their significance as a persistent global health challenge [1]. Together, these cancers account for approximately 40% of all cancer diagnoses each year [2]. According to the Global Cancer Observatory, there were 2.21 million new cases of lung cancer and 1.93 million cases of colorectal cancer reported in 2020, leading to 1.80 million and about 1.0 million deaths, respectively [3]. Tobacco consumption is the leading risk factor for lung cancer, accounting for the majority of global cases [4,5]. Moreover, several studies have suggested a possible pathological link between the two, where the systemic effects of one malignancy may increase susceptibility to the other [6]. The clinical co-occurrence of lung and colon cancers has been observed in multiple cases [7], reinforcing the need for early detection and precise histopathological diagnosis to improve treatment outcomes and survival rates.

Histopathological examination remains the gold standard for identifying and grading LC cancers; however, manual evaluation is labor-intensive, time-consuming, and susceptible to inter-observer variability. In recent years, deep learning (DL) and convolutional neural networks (CNNs) have shown remarkable promise in automating the analysis of histopathological images, achieving high accuracy in cancer detection and classification [8,9,10,11]. Several recent AI-based studies have reported strong performance on the LC25000 dataset, with accuracies ranging from 99.2% to 99.8% using hybrid feature extraction, multistage learning pipelines, or deep CNN architectures [12,13,14,15,16]. These results highlight the potential of deep learning for LC histopathology while also motivating the need for more lightweight and interpretable solutions. Despite these advances, many existing CNN frameworks still rely on large-scale architectures or complex optimization strategies that demand substantial computational resources and often function as “black boxes,” limiting their clinical interpretability and trustworthiness.

Nevertheless, opportunities remain for developing methods that are both computationally efficient and clinically interpretable. This study employs an artificial intelligence approach grounded in deep learning and builds upon prior AI-based histopathology research that has demonstrated strong performance in lung and colon cancer classification. In the era of explainable AI (XAI), transparency and interpretability are increasingly recognized as essential for the deployment of computer vision models in healthcare. Models must not only be accurate but also capable of providing visual reasoning that aligns with clinical understanding.

Building on this motivation, this study introduces DPCSE-Net, a lightweight dual-path convolutional neural network (CNN) enhanced with a Squeeze-and-Excitation (SE) attention mechanism for lung and colon histopathology classification. DPCSE-Net is designed to balance diagnostic accuracy, computational efficiency, and interpretability through multi-scale feature extraction and adaptive channel reweighting. The dual-path design captures fine-grained local details and global contextual information, while the SE attention mechanism dynamically highlights discriminative channels. Compared with existing histopathology models that often rely on large architectures, handcrafted feature fusion, or multistage processing pipelines, DPCSE-Net offers a lightweight and fully end-to-end design that achieves competitive accuracy with significantly lower computational cost and improved interpretability. To ensure transparency and clinical reliability, multiple explainable AI techniques, including Grad-CAM, attention heatmaps, and Integrated Gradients, are applied to visualize and interpret the model’s decision behavior. The framework is comprehensively evaluated against state-of-the-art deep learning models to validate its effectiveness in terms of both classification performance and interpretability.

The main contributions of this study are summarized as follows:

1. A lightweight, end-to-end dual-path CNN with asymmetric kernel scaling is proposed to enable efficient and effective multi-scale feature extraction from histopathological images.
2. A cross-path attention design is introduced by integrating a Squeeze-and-Excitation (SE) block after feature fusion to dynamically recalibrate and enhance discriminative multi-scale representations.
3. Multiple explainable AI (XAI) techniques, including Grad-CAM, attention heatmaps, and Integrated Gradients, are incorporated to ensure that the model’s focus aligns with clinically relevant tissue structures.
4. A computationally efficient training pipeline is developed to improve convergence stability and mitigate overfitting through adaptive learning strategies and callback mechanisms.

## 2. Related Work

The classification of lung and colon cancers from histopathological images has attracted considerable research attention due to its critical importance for early and accurate disease diagnosis. Numerous computer-aided diagnosis (CAD) frameworks have been proposed, leveraging convolutional neural networks (CNNs), feature fusion, and metaheuristic optimization to enhance diagnostic precision and computational efficiency [17,18].

Several studies have explored multi-CNN fusion to exploit complementary representations. Ijaz et al. [19] combined ResNet50 and EfficientNetB0 through serial feature integration and refined the resulting features using the Grey Wolf Optimization (GWO) algorithm, achieving 98.73% accuracy. Similarly, Attallah et al. [16] integrated lightweight CNNs (MobileNet, ResNet-18, and EfficientNetB0) with dual-layer feature extraction and statistical feature selection. Canonical Correlation Analysis (CCA) was employed for dimensionality reduction, while ANOVA and Chi-Squared tests identified the most discriminative features, achieving 99.8% accuracy on the LC25000 dataset.

To address computational constraints, several lightweight CNN-based solutions have been proposed. Mangal et al. [20] developed a custom CNN for classifying lung and colon histopathological images from the LC25000 dataset, achieving 97% accuracy for lung and 96% for colon cancer. Hasan et al. [12] introduced an end-to-end compact multiscale CNN enhanced with Grad-CAM and SHAP explainability, reaching 99.20% accuracy. Hadiyoso et al. [21] employed VGG16 with CLAHE-based contrast enhancement, improving performance to 98.96%. A hybrid model, ColonNet, proposed by Iqbal et al. [22], incorporated a Global–Local Pyramid Pattern (GLPP) for multiscale feature fusion and outperformed standard CNNs. Likewise, AlGhamdi et al. [23] combined ShuffleNet with a Deep Convolutional Recurrent Neural Network (DCRNN) and optimized it using the Al-Biruni Earth Radius (BER) and Coati Optimization Algorithm (COA), achieving 99.22% accuracy.

Further advancements have emerged through the fusion of deep and handcrafted features. Al-Jabbar et al. [13] combined VGG19 and GoogLeNet features with handcrafted descriptors such as Discrete Wavelet Transform (DWT), Local Binary Pattern (LBP), and Gray-Level Co-occurrence Matrix (GLCM). After dimensionality reduction using Principal Component Analysis (PCA), an Artificial Neural Network (ANN) achieved 99.64% accuracy and 100% specificity. Gowthamy and Ramesh [24] merged ResNet50, InceptionV3, and DenseNet features with a Kernel Extreme Learning Machine (KELM), attaining 99.0% accuracy. Lightweight CNN combinations were also investigated by Attallah et al. [14], who fused MobileNet, ShuffleNet, and SqueezeNet using Fast Walsh–Hadamard Transform (FWHT) and Discrete Wavelet Transform (DWT), achieving 99.6% accuracy with an SVM classifier.

In addition, hybrid optimization and ensemble-based frameworks have shown promise. Mengash et al. [25] developed a MobileNet–DBN pipeline optimized by the Marine Predator Algorithm (MPA) and enhanced with CLAHE preprocessing, yielding 99.28% accuracy on LC25000. Similarly, Singh and Singh [26] combined deep VGG16 and handcrafted LBP features, classifying them through an ensemble of SVM, Random Forest (RF), and Logistic Regression (LR), achieving 99.0% accuracy.

## 3. Materials and Methods

### 3.1. Dataset and Preprocessing

**Figure 1:**
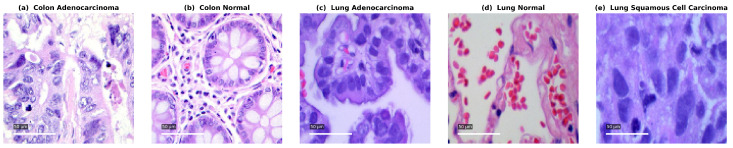
Sample histopathological images from the LC25000 dataset showing the five distinct classes: colon adenocarcinoma, colon benign tissue, lung adenocarcinoma, lung benign tissue, and lung squamous cell carcinoma.

The LC25000 dataset, introduced by Borkowski et al. [27], is a widely recognized benchmark for histopathological image analysis and has been extensively used in the classification of lung and colon cancers. It consists of 25,000 high-resolution histopathological images, each with a resolution of 768 × 768 pixels, divided into five balanced classes: colon adenocarcinoma, colon benign tissue, lung adenocarcinoma, lung benign tissue, and lung squamous cell carcinoma. Each class contains 5000 images, ensuring uniform representation and preventing class imbalance during model training. Representative samples from each class are shown in Figure 1 to illustrate the visual variation in histopathological appearance across categories.

All images were preprocessed through a consistent pipeline to ensure compatibility with the proposed DPCSE-Net architecture while preserving the original tissue morphology. Each image was resized to 128 × 128 × 3 pixels to match the network input dimensions and reduce computational complexity. Pixel intensity values were normalized to the range [0, 1] to standardize input scaling and promote stable convergence during training. The dataset was then stratified and split into training, validation, and testing subsets using a 60/20/20 ratio, maintaining class balance across all subsets.

### 3.2. The DPCSE-Net Architecture

**Figure 2:**
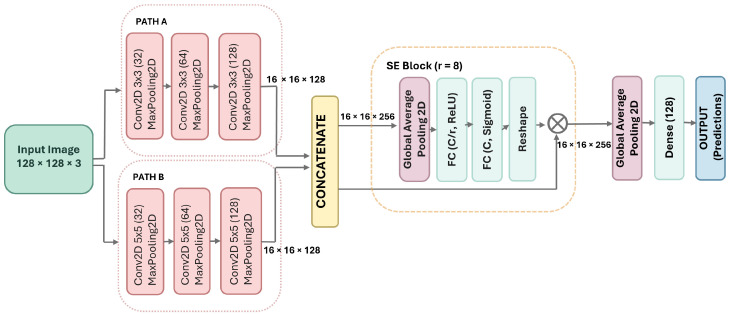
DPCSE-Netarchitecture integrating dual-path convolutions and an SE attention block, where FC(C/r, ReLU) and FC(C, Sigmoid) denote channel reduction and restoration layers before classification.

The proposed DPCSE-Net is a lightweight dual-path convolutional neural network (CNN) designed for accurate and efficient classification of lung and colon histopathological images. The model captures both local cellular textures and broader morphological structures through parallel convolutional paths, followed by channel-wise feature recalibration using a Squeeze-and-Excitation (SE) block. An overview of the network architecture is presented in Figure 2.

#### 3.2.1. Input

Each histopathological image from the LC25000 dataset is resized to 128 × 128 × 3 pixels before being fed into the network. This compact input size minimizes computational cost while preserving essential morphological details required for discrimination between tissue types.

#### 3.2.2. Dual-Path Processing

The input image is processed simultaneously through two independent convolutional branches, **Path A** and **Path B**, designed to extract complementary spatial information.

- **Path A** employs 3 × 3 convolutional filters in three consecutive blocks with 32, 64, and 128 filters, respectively. Each convolutional block is followed by a MaxPooling2D layer to progressively reduce spatial dimensions while retaining key local features.
- **Path B** utilizes 5 × 5 convolutional filters in three consecutive blocks with 32, 64, and 128 filters, respectively. Each convolutional block is followed by a MaxPooling2D layer to capture broader spatial context while progressively reducing spatial dimensions.

This dual-path structure allows the model to learn both fine-grained cellular patterns and large-scale contextual information within the same forward pass.

#### 3.2.3. Feature Concatenation

The outputs of Path A and Path B are concatenated along the channel dimension to form a unified multiscale feature representation. This fusion integrates local and global spatial cues, enhancing the model’s ability to distinguish subtle differences between benign and malignant tissue regions.

#### 3.2.4. Squeeze-and-Excitation Block

To enhance the representational capacity of the fused feature maps, DPCSE-Net incorporates a Squeeze-and-Excitation (SE) block, originally proposed by Hu et al. [28]. The SE mechanism performs channel-wise attention by modeling dependencies between feature channels, enabling the network to prioritize informative features while suppressing less relevant ones.

The SE block operates in two stages: squeeze and excitation. In the squeeze stage, global average pooling (GAP) compresses each feature map into a single representative value, producing a channel descriptor that captures global spatial information:

(1) $$z_{c}=\frac{1}{H\times W}\sum_{i=1}^{H}\sum_{j=1}^{W}X_{c}\left(i,j\right),$$

where $X_{c}\left(i,j\right)$ denotes the activation at spatial position (i,j) in the *c*-th feature channel, and *H* and *W* represent the height and width of the feature map.

In the excitation stage, two fully connected layers learn nonlinear channel interdependencies and generate adaptive attention weights using a sigmoid activation:

(2) $$s=\sigma \left(W_{2}\;\mathrm{ReLU}\left(W_{1}\;z\right)\right),$$

where $z={\left[z_{1},z_{2},\ldots ,z_{C}\right]}^{T}$ is the squeezed channel vector, $W_{1}\in R^{\frac{C}{r}\times C}$ and $W_{2}\in R^{C\times \frac{C}{r}}$ are the learnable weights of the two fully connected layers, *r* is the reduction ratio, ReLU(·) denotes the Rectified Linear Unit activation, and σ(·) is the sigmoid function that normalizes the weights between 0 and 1.

For compact representation, the complete squeeze and excitation operation can be expressed as

(3) $$\tilde{X}=\sigma \left(W_{2}\;\mathrm{ReLU}\left(W_{1}\;\mathrm{GAP}\left(X\right)\right)\right)⊙X,$$

where ⊙ denotes element-wise (channel-wise) multiplication. This formulation describes the end-to-end flow of the SE mechanism, from global context extraction to adaptive channel recalibration, allowing DPCSE-Net to emphasize diagnostically meaningful regions while maintaining computational efficiency.

In DPCSE-Net, the SE block is positioned immediately after the feature concatenation stage to refine the joint feature representations extracted by the dual convolutional paths. This adaptive recalibration enables the network to highlight diagnostically important visual cues such as variations in nuclear morphology, glandular structures, and textural irregularities while reducing background noise and staining variability. By introducing this lightweight attention mechanism, DPCSE-Net achieves stronger discriminative power and improved robustness without increasing model complexity.

#### 3.2.5. Classification and Output Layer

After channel-wise attention refinement, the feature map undergoes global average pooling (GAP) to reduce spatial dimensions while retaining the most discriminative activations. The resulting feature vector is passed through a dense layer with 128 units and nonlinear activation, followed by a final dense layer with 5 units and softmax activation to output class probabilities corresponding to the five diagnostic categories: colon adenocarcinoma, colon benign tissue, lung adenocarcinoma, lung benign tissue, and lung squamous cell carcinoma.

### 3.3. Explainability and Model Interpretation

To enhance interpretability and clinical transparency, the proposed DPCSE-Net framework integrates several explainable artificial intelligence (XAI) techniques that visualize how the network makes diagnostic decisions. Three complementary methods were employed to provide spatial, channel-wise, and pixel-level interpretability: Gradient-weighted Class Activation Mapping (Grad-CAM), SE Attention Heatmaps, and Integrated Gradients. Each method reveals a distinct aspect of DPCSE-Net’s decision process, as described below.

#### 3.3.1. Gradient-Weighted Class Activation Mapping (Grad-CAM)

Grad-CAM highlights the most discriminative regions contributing to a specific class prediction. Proposed by Selvaraju et al. [29], this method generates class-discriminative heatmaps by computing the gradient of the target class score $y^{c}$ with respect to the feature maps $A^{k}$ of a convolutional layer:

(4) $$\alpha_{k}^{c}=\frac{1}{Z}\sum_{i}\sum_{j}\frac{\partial y^{c}}{\partial A_{ij}^{k}},$$

(5) $$L_{\mathrm{GradCAM}}^{c}=\mathrm{ReLU}\left(\sum_{k}\alpha_{k}^{c}A^{k}\right),$$

where $\alpha_{k}^{c}$ represents the importance of feature map *k* for class *c*, $A^{k}$ denotes the activation map, and *Z* is the number of spatial positions. The ReLU ensures that only features positively influencing the class are visualized. This enables the identification of diagnostically relevant regions such as nuclei clusters or glandular boundaries.

#### 3.3.2. SE Attention Heatmaps

The SE attention mechanism [28], which is integrated within DPCSE-Net, provides intrinsic interpretability by highlighting the relative importance of feature channels. Instead of relying on external post-hoc attention methods, the SE module inherently learns to recalibrate feature responses during training. The resulting channel-wise attention weights, obtained after the feature fusion stage, are visualized as attention heatmaps that indicate which channels contribute most strongly to each classification decision. These visualizations show how DPCSE-Net emphasizes diagnostically relevant patterns such as glandular organization, nuclear density, and textural variations while reducing the influence of background structures.

#### 3.3.3. Integrated Gradients

Integrated Gradients provide pixel-level attribution to quantify how individual pixels influence the model prediction. Proposed by Sundararajan et al. [30], this method integrates gradients along a path from a baseline input $x^{\prime }$ to the actual input *x*:

(6) $${\mathrm{IG}}_{i}\left(x\right)=\left(x_{i}-x_{i}^{\prime }\right)\times \int_{0}^{1}\frac{\partial F(x^{\prime }+\alpha \left(x-x^{\prime }\right))}{\partial x_{i}}\;d\alpha ,$$

where F(·) denotes the model output and ${\mathrm{IG}}_{i}\left(x\right)$ quantifies the contribution of pixel *i* to the final decision. This approach complements Grad-CAM and SE attention by providing fine-grained, pixel-level interpretability that supports detailed clinical assessment.

#### 3.3.4. Explainability Pipeline Summary

The complete XAI workflow applied to DPCSE-Net is summarized in Algorithm 1. Grad-CAM highlights spatial regions that influence classification outcomes, SE attention captures channel relevance through intrinsic attention weights, and Integrated Gradients quantify pixel-level contributions. This combined strategy provides spatial, channel-wise, and attribution-based explanations that enhance both the interpretability and transparency of DPCSE-Net. Quantitative and qualitative visualization results are presented in Section 4.
**Algorithm 1** Explainability Pipeline for DPCSE-Net**Require:** Input image *x*, trained DPCSE-Net model *F*
  1:Compute feature maps $A^{k}$ and class score $y^{c}=F\left(x\right)$  2:**Grad-CAM:** Compute importance weights $\alpha_{k}^{c}$ and generate the spatial heatmap $L_{\mathrm{GradCAM}}^{c}$ to visualize class-discriminative regions  3:**SE-Attention:** Extract and normalize channel-wise attention weights *s* from the SE module to form $H_{\mathrm{SE}}$, indicating the most informative feature channels  4:**Integrated Gradients:** Calculate the pixel-level attribution map IG(x) by integrating gradients from a baseline $x^{\prime }$ to the input image *x*  5:Return the visualization set $\left\{L_{\mathrm{GradCAM}}^{c},\;H_{\mathrm{SE}},\;\mathrm{IG}\left(x\right)\right\}$


Together, these interpretability methods provide a multi-level understanding of DPCSE-Net’s reasoning process. Grad-CAM highlights localized diagnostic regions, SE attention visualizes learned channel importance, and Integrated Gradients reveal pixel-wise attributions. This multi-level explainability ensures that DPCSE-Net’s predictions remain transparent, interpretable, and aligned with clinically meaningful visual cues.

### 3.4. Experimental Setup

#### 3.4.1. Training Setup

All experiments were implemented in Python 3.12.12 using the TensorFlow 2.19.0 and Keras 3.10.0 deep learning frameworks. Model training was conducted in the Google Colab Pro environment with GPU acceleration, which provided efficient computation for large-scale histopathological images and significantly reduced training time through parallelized processing.

Model performance was assessed using standard evaluation metrics commonly adopted in medical image classification, including accuracy, precision, recall, and F1-score [31,32]. These metrics are defined as follows:

(7) $$\mathrm{Accuracy}=\frac{TP+TN}{TP+TN+FP+FN},$$

(8) $$\mathrm{Precision}=\frac{TP}{TP+FP},\;\mathrm{Recall}=\frac{TP}{TP+FN},\;F1\text{-}\mathrm{score}=2\times \frac{\left(\mathrm{Precision}\times \mathrm{Recall}\right)}{\mathrm{Precision}+\mathrm{Recall}},$$

where TP, TN, FP, and FN denote true positives, true negatives, false positives, and false negatives, respectively. Precision quantifies the proportion of correctly identified positive cases among all predicted positives, whereas recall measures the model’s ability to identify all actual positive cases. The F1-score represents the harmonic mean of precision and recall, providing a balanced measure of sensitivity and specificity. These metrics collectively offer a comprehensive evaluation of model performance, ensuring both accuracy and reliability in medical decision-support scenarios.

#### 3.4.2. Training Configuration

Model training was performed using an optimized input pipeline implemented with the TensorFlow API, which employed batching (32 samples per batch), shuffling (buffer size = 1000), and prefetching to ensure smooth data loading and efficient GPU utilization. The model was compiled using the Nadam optimizer [33] with a learning rate of 0.001, and the categorical cross-entropy loss function [34] was adopted for multi-class classification. Accuracy was monitored as the primary performance metric during both training and validation. All hyperparameters, including the optimizer configuration, learning rate, and number of epochs, were tuned empirically to balance accuracy and generalization performance.

To ensure robust convergence and mitigate overfitting, several callback mechanisms were integrated. Early stopping was employed to halt training when the validation loss failed to improve for five consecutive epochs, with the best model weights automatically restored. A ReduceLROnPlateau scheduler decreased the learning rate by a factor of 0.5 after three stagnant epochs, while an additional exponential decay scheduler gradually reduced the learning rate after the tenth epoch to stabilize late-stage optimization. Model checkpointing was also utilized to automatically save the model exhibiting the lowest validation loss for subsequent evaluation.

The network was trained for up to 50 epochs, which consistently ensured efficient convergence and stable performance across all experimental runs.

## 4. Results

### 4.1. Quantitative Evaluation and Ablation Analysis

#### 4.1.1. Overall Performance on the LC25000 Dataset

The proposed DPCSE-Net, combining dual-path convolutional feature extraction with Squeeze-and-Excitation (SE) attention, achieved outstanding quantitative performance on the LC25000 dataset. Across all diagnostic categories, the model attained an accuracy, precision, recall, and F1-score of 99.88%, demonstrating exceptional balance between sensitivity and specificity.

**Figure 3:**
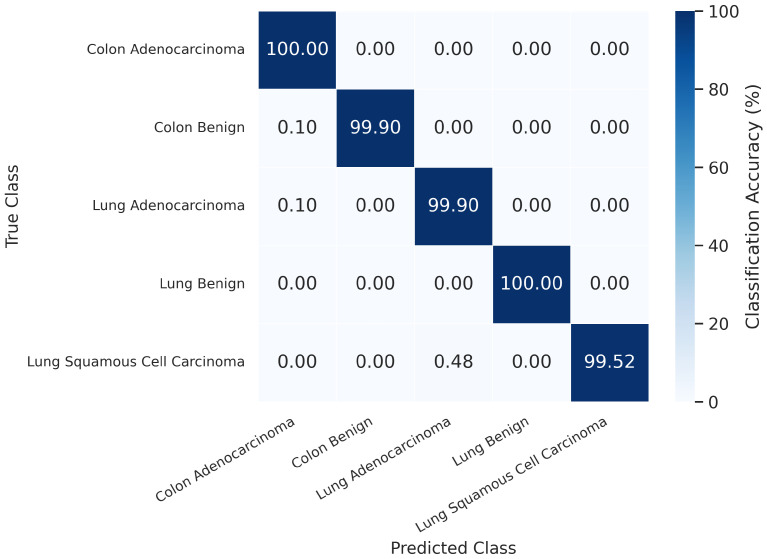
Normalized confusion matrix illustrating class-wise accuracy of DPCSE-Net on the LC25000 test set. Each cell represents the percentage of correct predictions per class, with values above 99.5% across all categories. Minimal confusion was observed only between morphologically similar carcinoma subtypes.

To provide a clearer depiction of per-class accuracy, Figure 3 presents the normalized confusion matrix, where each cell value represents the classification accuracy in percentage for a given class. All five categories achieved nearly perfect recognition, with diagonal values exceeding 99.5%. Minor confusion was observed only in the *lung squamous cell carcinoma* (SCC) class, where a few samples (0.48%) were misclassified as *lung adenocarcinoma*, likely due to overlapping morphological structures and staining patterns. Such results validate the strong discriminative capacity of DPCSE-Net in capturing fine-grained textural differences among histopathological subtypes.

**Figure 4:**
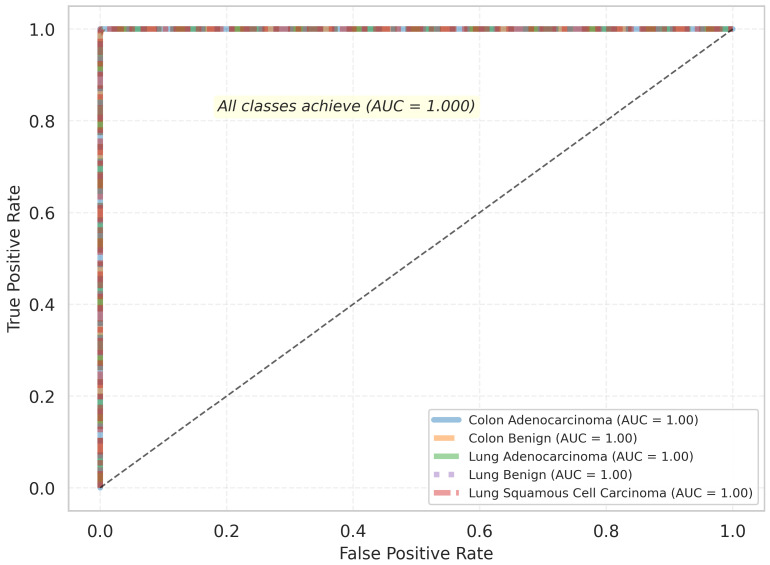
Receiver Operating Characteristic (ROC) curves of DPCSE-Net for the five histopathological classes in the LC25000 dataset. All classes achieved an AUC of 1.00, indicating near-perfect sensitivity and specificity.

The Receiver Operating Characteristic (ROC) analysis, shown in Figure 4, further demonstrates these findings. Each class achieved an Area Under the Curve (AUC) value of 1.00, reflecting near-perfect sensitivity and specificity. The steep, top-left trajectory of the ROC curves confirms the model’s ability to minimize false positives while maintaining high true positive rates, illustrating the effectiveness of its multiscale dual-path architecture with channel-wise SE attention.

#### 4.1.2. Ablation Study

**Figure 5:**
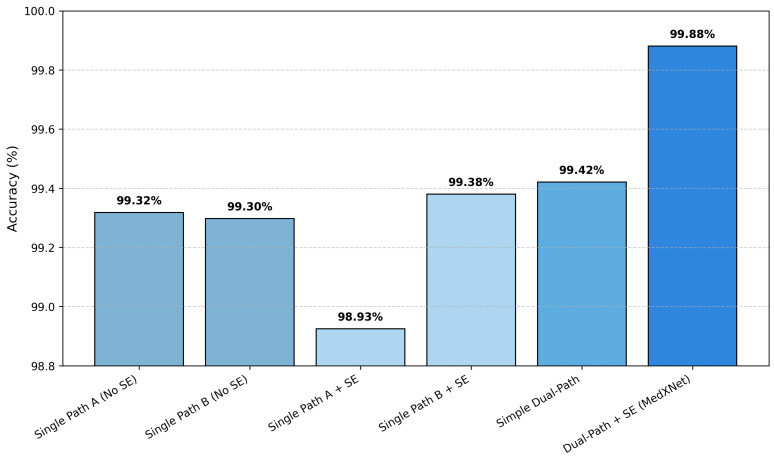
Accuracy comparison of DPCSE-Net variants on the LC25000 dataset. The full dual-path configuration with SE attention achieved the highest accuracy (99.88%), confirming the complementary benefits of multiscale feature extraction and channel-wise recalibration.

An ablation study was conducted to evaluate the contribution of each architectural component of the proposed DPCSE-Net, namely the dual-path design and the Squeeze-and-Excitation (SE) attention mechanism. Multiple model variants were implemented to isolate and assess the effect of each component on classification performance. The results are summarized in Table 1 and visualized in Figure 5.

The single-path variants achieved strong baseline performance, with accuracies between 98.93% and 99.32%, indicating that each convolutional stream independently learns discriminative morphological features. Incorporating SE attention within a single path yielded a minor improvement in Path B (+0.08%), showing that adaptive channel recalibration enhances feature selectivity in deeper receptive fields.

When both paths were combined without attention, the accuracy increased to 99.42%, confirming that fusing multiscale features enriches contextual representation. The complete DPCSE-Net, which integrates the dual-path architecture with SE attention, achieved the highest accuracy and F1-score of 99.88%, marking a performance gain of +0.46% over the dual-path baseline and +0.95% over single-path variants.

These results demonstrate that both components contribute cooperatively to DPCSE-Net’s effectiveness. The dual-path structure captures diverse spatial scales, while the SE attention mechanism adaptively enhances diagnostically relevant channels.

### 4.2. Computational Efficiency Analysis

To complement the quantitative evaluation, we assessed the computational efficiency of the proposed DPCSE-Net. The key characteristics, including parameter count, floating point operations, and inference speed, are summarized in Table 2. The model contains 287,525 trainable parameters with no non-trainable components, resulting in a compact size of 1.10 MB.

The total computational cost is 0.988 GFLOPs for a 128×128×3 input, with the detailed breakdown provided in Table 3. Path B contributes 0.649 GFLOPs (65.7%), Path A contributes 0.339 GFLOPs (34.3%), and the SE and classifier layers together account for less than 0.02% of the total.

These results show that DPCSE-Net maintains a lightweight architecture and delivers fast inference suitable for high-throughput histopathology workflows.

### 4.3. Comparative Analysis with Existing Methods

The comparative results in Table 4 show that the proposed DPCSE-Net achieves state-of-the-art performance on the LC25000 dataset, matching or surpassing leading frameworks such as those of Al-Jabbar et al. [13] and Attallah [16]. While previous studies obtained high accuracy through handcrafted feature fusion, statistical feature selection, or multistage ensemble architectures, DPCSE-Net achieves comparable precision and sensitivity within a single lightweight, end-to-end network.

The dual-path design captures both fine-grained cellular details and broader tissue context, and the integrated SE attention mechanism enhances discriminative feature learning without introducing notable computational overhead. Model-size information has also been incorporated into the comparison to highlight the compact nature of DPCSE-Net relative to existing approaches, with “–” indicating cases where parameter counts or FLOPs were not reported.

This balance between accuracy, architectural simplicity, and computational efficiency underscores the suitability of DPCSE-Net for real-time histopathological analysis and practical clinical deployment.

### 4.4. Explainability and Visual Interpretation

**Figure 6:**
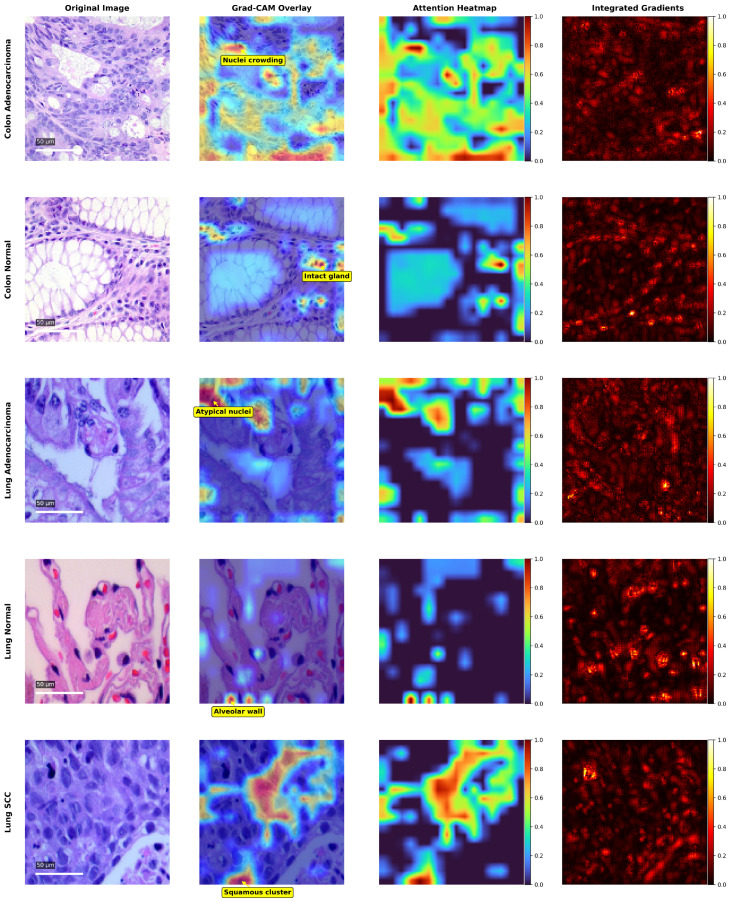
Explainability visualization of DPCSE-Net across lung and colon histopathological classes. Columns correspond to the original image, Grad-CAM overlay, SE attention heatmap, and Integrated Gradients attribution map. Annotated arrows highlight clinically relevant regions of model attention.

Representative visual results are presented in Figure 6, illustrating how the proposed DPCSE-Net model interprets and localizes diagnostically relevant features across different lung and colon histopathological classes. Each row corresponds to a specific diagnostic category, while the columns display the original image, Grad-CAM overlay, SE attention heatmap, and Integrated Gradients attribution map. To further enhance clinical interpretability, arrows and brief labels were added to highlight key histopathological structures that align with the model’s strongest activation regions. These visualizations collectively provide spatial, channel-wise, and pixel-level insights into the model’s reasoning process.

Gradient-weighted Class Activation Mapping (Grad-CAM) highlights the spatial regions that most strongly contribute to each classification outcome. The generated overlays reveal that DPCSE-Net concentrates on morphologically meaningful regions such as dense nuclei clusters, glandular borders, and cytoplasmic textures indicative of malignancy. This confirms that the model captures discriminative histopathological structures rather than relying on color variations or background artifacts.

SE Attention heatmaps, derived from the embedded Squeeze-and-Excitation module, visualize how channel-wise activations are adaptively re-weighted during inference. Higher responses were consistently observed in channels representing nuclei-rich or irregular tissue regions, confirming the module’s ability to emphasize the most informative feature maps while suppressing irrelevant ones.

Integrated Gradients provide a complementary, pixel-level attribution analysis by quantifying the contribution of each pixel to the model’s output. The resulting maps closely align with Grad-CAM and SE attention patterns, reinforcing the consistency of DPCSE-Net’s learned representations and validating its focus on biologically relevant structures.

Overall, the alignment among Grad-CAM, SE attention, and Integrated Gradients demonstrates that DPCSE-Net bases its decisions on clinically interpretable features that pathologists commonly use in diagnosis. This consistency across interpretability methods enhances confidence in the model’s transparency, diagnostic reliability, and potential for practical integration in computational pathology workflows.

The observed focus regions align with the architectural intent of DPCSE-Net. The dual-path design facilitates multi-scale perception, where Path A captures fine-grained nuclear textures, while Path B encodes broader glandular context. The SE attention module subsequently refines these fused representations by up-weighting channels corresponding to diagnostically relevant morphological features. This correspondence between architectural structure and visualization outcomes further validates the model’s design rationale.

## 5. Discussion

### 5.1. Summary of Findings

The experimental results demonstrate that the proposed DPCSE-Net framework achieves a well-balanced trade-off between accuracy, interpretability, and computational efficiency for lung and colon histopathological image classification. The dual-path architecture enables the simultaneous extraction of fine-grained cellular textures and broader tissue morphology, while the embedded SE attention mechanism adaptively enhances the most discriminative feature channels. This interaction allows DPCSE-Net to deliver superior performance compared with recent state-of-the-art models on the LC25000 dataset, achieving 99.88% accuracy and F1-score with substantially lower architectural complexity.

### 5.2. Ablation and Explainability Insights

The ablation study confirmed the complementary contribution of both architectural components. Removing either the dual-path structure or the SE block resulted in a measurable decline in classification performance, highlighting their cooperative role in robust feature learning. Furthermore, the explainability analysis demonstrated that DPCSE-Net consistently focuses on diagnostically relevant regions such as glandular structures, nuclear clusters, and cytoplasmic boundaries, while effectively suppressing background and staining artifacts. This strong correspondence between quantitative accuracy and qualitative interpretability reinforces confidence in the model’s reliability for clinical decision support. Although LC25000 exhibits relatively uniform staining, real-world histopathology often presents substantial stain variability across centers and scanners. The multi-scale dual-path design may offer inherent robustness to such variation, yet dedicated multi-center benchmarking remains necessary to fully assess generalizability.

### 5.3. Comparison with Previous Studies

Recent deep learning studies on the LC25000 dataset have achieved high accuracy through hybrid feature fusion, multistage learning pipelines, or large-scale CNN architectures [12,13,14,15,16]. While effective, these methods often introduce significant computational complexity and limited interpretability.

In contrast, DPCSE-Net attains comparable diagnostic performance within a lightweight, end-to-end design. The dual-path structure efficiently captures multiscale features, and the integrated SE attention mechanism strengthens discriminative learning without increasing architectural depth. Moreover, the inclusion of Grad-CAM, SE attention heatmaps, and Integrated Gradients provides intrinsic interpretability, offering clearer insight into the model’s decision process than traditional “black-box” CNNs.

These characteristics highlight DPCSE-Net as a compact, efficient, and transparent alternative to existing approaches, supporting its suitability for practical and clinically oriented histopathology workflows.

### 5.4. Limitations

Despite these promising results, several aspects warrant further investigation. The current evaluation was limited to the LC25000 dataset, which, although diverse, may not fully capture the histopathological variability encountered across clinical centers and staining protocols. Future studies should integrate robust stain normalization techniques and conduct multi-center validation to ensure generalizability. Moreover, real-world clinical images often exhibit artifacts, variable image quality, and borderline cases not well represented in curated datasets. Another limitation is that the LC25000 dataset consists of fixed-size image patches rather than whole-slide images (WSIs), which restricts the evaluation of model performance under real clinical conditions. This limitation prevents the assessment of robustness to tissue folds, debris, slide preparation variability, heterogeneous tumor margins, and other artifacts that routinely appear in diagnostic workflows.

### 5.5. Clinical Considerations and Future Work

The deployment of AI diagnostic systems also requires careful consideration of ethical and regulatory dimensions. While DPCSE-Net’s explainability features improve transparency, the model should serve as an assistive tool under pathologist supervision rather than an autonomous diagnostic system. Ensuring regulatory compliance, data privacy, and extensive clinical validation across diverse populations remains essential for clinical adoption. Future work will include pathologist-in-the-loop evaluations to assess the practical utility of the explainability features in routine diagnostic workflows.

### 5.6. Overall Implications

Overall, the findings demonstrate that integrating lightweight multiscale feature extraction with embedded channel attention and interpretability mechanisms yields a powerful yet transparent framework for cancer histopathology classification. DPCSE-Net not only achieves strong empirical performance but also establishes a practical foundation for developing deployable and trustworthy AI systems in digital pathology. The insights gained from this study may guide the design of future architectures that jointly emphasize diagnostic accuracy, efficiency, and interpretability in medical imaging applications.

## 6. Conclusions

The aim of this study was to develop a lightweight, accurate, and interpretable deep learning framework for the automated classification of lung and colon histopathological images. This study introduced DPCSE-Net, a lightweight dual-path convolutional neural network enhanced with a Squeeze-and-Excitation (SE) attention mechanism for automated classification of lung and colon histopathological images. By integrating multiscale feature extraction with adaptive channel recalibration, the framework achieves a strong balance between accuracy, interpretability, and computational efficiency. The proposed model achieved a classification accuracy of 99.88% on the LC25000 dataset, demonstrating competitive or superior performance compared with recent state-of-the-art methods while requiring fewer parameters and lower computational cost. Furthermore, integrated explainability techniques, including Grad-CAM, SE attention heatmaps, and Integrated Gradients, provided transparent visual evidence that the model focuses on diagnostically meaningful regions, reinforcing its clinical reliability. Future work will extend DPCSE-Net to multi-center datasets with varied staining conditions and include pathologist-in-the-loop validation to enhance generalizability and clinical adoption.

## Figures and Tables

**Table 1:** Ablation analysis of DPCSE-Net components on the LC25000 dataset.

Model Variant	Accuracy (%)	Precision (%)	Recall (%)	F1-Score (%)
Single Path A (No SE)	99.32	99.32	99.32	99.32
Single Path B (No SE)	99.30	99.30	99.30	99.30
Single Path A + SE	98.93	98.95	98.93	98.92
Single Path B + SE	99.38	99.38	99.38	99.38
Simple Dual-Path (No SE)	99.42	99.43	99.42	99.42
Dual-Path + SE (Full DPCSE-Net)	**99.88**	**99.88**	**99.88**	**99.88**

**Table 2:** Computational characteristics of DPCSE-Net.

Metric	Value	Description
Total parameters	287,525	Trainable only
Model size	1.10 MB	Float32 representation
FLOPs per inference	0.988 GFLOPs	128 × 128 × 3 input
Inference time	2.41 ms	Per image on GPU
Throughput	415 images/s	Batch size of 32

**Table 3:** Detailed FLOP breakdown by functional block.

Functional Block	GFLOPs	Percentage
Path A	0.339	34.28%
Path B	0.649	65.70%
Squeeze-and-Excitation	0.000098	0.01%
Classifier	0.000067	0.01%
Total	0.988	100.00%

**Table 4:** Comparative performance of DPCSE-Net against representative state-of-the-art methods on the LC25000 dataset. The proposed framework achieves superior or comparable accuracy while maintaining a compact, end-to-end design. Entries marked “–” indicate that model size or parameter information was not reported in the original publications.

Authors (Year)	Accuracy (%)	Sensitivity (%)	Specificity (%)	Precision (%)	F1-Score (%)	Model Size/Parameters
Hasan et al. (2024) [12]	99.20	99.36	99.16	99.16	–	1.10 M parameters
Al-Jabbar et al. (2023) [13]	99.64	99.85	**100.00**	**100.00**	–	–
Attallah et al. (2022) [14]	99.60	99.60	99.90	99.60	99.60	–
Alsubai (2024) [15]	99.88	99.42	99.46	99.76	–	–
Attallah (2025) [16]	99.78	99.78	99.95	99.78	99.78	–
**DPCSE-Net (Proposed)**	**99.88**	**99.88**	99.88	99.88	**99.88**	287,525 parameters

## Data Availability

The dataset analyzed in this study (LC25000) is publicly available at https://www.kaggle.com/datasets/andrewmvd/lung-and-colon-cancer-histopathological-images (accessed on 14 April 2025). All codes used for model implementation, training, and visualization are available from the author upon request.

## Abbreviations

The following abbreviations are used in this manuscript:

AI	Artificial Intelligence
CAD	Computer-Aided Diagnosis
CNN	Convolutional Neural Network
SE	Squeeze and Excitation
XAI	Explainable Artificial Intelligence
Grad-CAM	Gradient-weighted Class Activation Mapping
GAP	Global Average Pooling
ReLU	Rectified Linear Unit
AUC	Area Under the Curve
ROC	Receiver Operating Characteristic
TP	True Positive
TN	True Negative
FP	False Positive
FN	False Negative
ANN	Artificial Neural Network
SVM	Support Vector Machine
